# MicroRNA-145 overexpression inhibits neuroblastoma tumorigenesis *in vitro* and *in vivo*

**DOI:** 10.1080/21655979.2020.1729928

**Published:** 2020-02-21

**Authors:** Jing Zhao, Kai Zhou, Liang Ma, Huanyu Zhang

**Affiliations:** a Department of Pediatric Surgery, Hospital of Qingdao University, Qingdao, Shandong, China; b Urology, Hospital of Qingdao University, Qingdao, Shandong, China; c Child Health Division, Hospital of Qingdao University, Qingdao, Shandong, China

**Keywords:** Neuroblastoma, miR-145, MTDH, apoptosis, tumorigenesis

## Abstract

Neuroblastoma (NB) is responsible for 15% of all childhood cancer deaths. Despite advances in treatment and disease management, the overall 5-year survival rates remain poor in high-risk disease (25–40%). It is well known that miR-145 functions as a tumor suppressor in several types of cancer. However, the impact of miR-145 on NB is still ambiguous. Our aim was to investigate the potential tumor suppressive role and mechanisms of miR-145 in high-risk neuroblastoma. Expression levels of miR-145 in tissues and cells were determined using RT-qPCR. The effect of miR-145 on cell viability was evaluated using MTT assays, apoptosis levels were determined using TUNEL staining, and the MTDH protein expression was determined using western blot and RT-PCR. Luciferase reporter plasmids were constructed to confirm direct targeting for MTDH. **The results showed** that miR-145 expression was significantly lower in high-risk MYCN amplified (MNA) tumors and low miR-145 expression was associated with worse EFS and OS in our cohort. Over-expression of miR-145 reduced cell viability and increased apoptosis in SH-SY-5Y cells. We identified MTDH as a direct target for miR-145 in SH-SY-5Y cells. Targeting MTDH has the similar results as miR-145 overexpression. Our findings suggest that low miR-145 expression was associated with poor prognosis in patients with NB, and the overexpression of miR-145 inhibited NB cells growth by down-regulating MTDH, thus providing a potential target for the development of microRNA-based approach for NB therapy.

## Introduction

Neuroblastoma (NB) accounts for around 15% of pediatric oncology deaths. Most primary **NBs** arise within the abdomen, in particular from the adrenal gland, although tumors can arise anywhere along the sympathetic nervous system [1]. **NB is** clinically diverse, ranging from spontaneously regressing to metastatic and treatment-resistant disease. NB patients are classified according to pretreatment risk groups: very low, low, intermediate and high risk. NB is characterized by extensive clinical heterogeneity, illustrated by different clinical evolutions ranging from spontaneous regression to aggressive disease [2]. Therefore, prognostic markers that accurately differentiate low- and high-risk patients are essential.

Treatment strategies for high-risk disease include high-dose chemotherapy [3], surgery [4], radiotherapy [5], anti-GD2 immunotherapy [6] and targeting therapy [7,8]. Despite recent FDA approval of anti-GD2 antibody-based immunotherapy for high-risk NB patients in first remission, the cure rates remain less than 50% accompanied by significant morbidity in survivors [9]. Thus, improvements in survival rates for children with high-risk NB are dependent on novel treatment approaches.

MicroRNAs (miRNAs) are a class of short non-coding RNAs that have emerged as significant epigenetic regulators of cellular functions, predominantly through silencing of their target genes via direct complementary mRNA 3′UTR base pairing [10]. Dysregulation of miRNAs has been reported in numerous cancers where individual miRNAs behave in an oncogenic or tumor suppressor manner [11–14]. To date, several profiling studies have reported that miRNAs are associated with clinical outcome in NB [15] and specific miRNAs have been identified to regulate key processes such as apoptosis, differentiation, cell proliferation and cell invasiveness in NB [16–18].

MiR-145, located in a fragile region of chromosome 5q has been reported to play a tumor suppressor role in a variety of other cancers, including NB [19]. As a cancer suppressor gene, it inhibits proliferation, invasion, and metastasis of tumor cells, increases sensitivity to chemotherapeutic drugs, and regulates the development and progression of tumors [20,21]. Decreased miR-145 expression has reported in a wide range of tumors including prostate, bladder, colon, ovarian, and esophageal cancers, suggesting a potential role in disease pathology. In-depth studies suggest that miR-145 could be an ideal marker for diagnostic and prognostic evaluations of tumors, and that it may also represent a novel target for cancer treatment [21,22]. However, the role of miR-145 in NB is still unclear.

In the present study, we aimed to evaluate the relation of miR-145 expression and potential clinical signiﬁcance of miR-145 in NB. We further deﬁne the tumor suppressive function of miR-145 in NB, by elucidating the underlying mechanisms for such function of the miR-145-mediated tumor suppressive pathway in neuroblastoma development.

## Materials and methods

### Cell culture

Human NB cell lines SH-SY-5Y were obtained from the American Type Culture Collection **(ATCC, Shanghai, China)**. The identities of SH-SY-5Y were verified by small tandem repeat profiling at the Dana-Farber Cancer Institute and the Broad Institute. The cell lines were maintained in DMEM (Cellgro) supplemented with 10% fetal bovine serum (Sigma-Aldrich) and 1% penicillin-streptomycin with glutamine (Cellgro), and was cultured at **37°C** in a humidified atmosphere containing 5% CO_2_.

### Patient materials

Between January 2012 and December 2018, a total of 130 subjects aged 0–18 years with previously untreated neuroblastoma (NB) were diagnosed in the affiliated hospital of Qingdao university. NB tumor tissues obtained from these patients were immediately (< 60 min) fresh frozen at surgery. Patients’ clinical records were reviewed to obtain details. Relevant informed consent was obtained according to the ethical approval from the affiliated hospital of Qingdao university Research Ethics Committee (approval no 2010/11/06).

### PremiRs and antagomiRs transfection

PremiRs (Ambion, Applied Biosystems) or antagomiR oligonucleotides (Eurogentec) were transfected (100 μM) using siIMPORTER (Millipore) according to the manufacturer’s procedures. Negative control premiR (Ambion) or scrambled antagomiR were transfected as controls. Chemically modiﬁed antisense oligonucleotides (antagomiR) have been used to inhibit miR expression in vitro. The sequences of antagomiR-145 used is as follows: 5′-AGGGATTCCTGGGAAAACTGGAC-3′. The scrambled antimiR sequence was 5′-CAGCTGAAGTAAATACCGACCAG-3′.

### Lentivirus-based miRNA precursor constructs

PMIRNA1 – miR-145 and pMIRNA1 – Empty Vector were provided in E. coli bacterial stock form plated on LB-carbenicillin at 50 µg/ml (System Biosciences, Mountain View, CA). The viral particles were obtained with the protocol from the System Biosciences User Manual, using the 293-TN cell line and pPACKH1 Lentivector Packaging Kit (System Biosciences). Virus pellets were resuspended in DMEM and stored in cryovials at −80°C until use.

### MiR-145 lentivirus infection

SH-SY-5Y cells were infected with the miR-145 lentivirus with an efficiency of approximately 50% as determined by green fluorescent protein measurement by flow cytometry. Empty lentivirus (LVEV, lentivirus empty vector) was used as a negative control for the experiments.

### Luciferase assay

Empty vector or miR-145 and luciferase reporter comprising 3′-UTR of MTDH wild-type or mutant fragment (**GeneChem, Shanghai, China**) were co-transfected using Lipofectamine 2000 (**Invitrogen/Thermo Fisher Scientific**) into 293T cells (**ATCC, Shanghai, China**) cultured in a 96-well plate. Following 72 h of transfection, the cells were harvested and luciferase activity was measured the chemiluminescence using the dual-luciferase reporter assay (**normalization with *Renilla* luciferase activity, Promega, Madison, WI, USA**) according to the manufacturer’s instructions.

### Transient siRNA transfection

Cells were grown to confluence in 6-cm tissue culture plates, and transfected with one of the four MTDH-specific siRNA oligos at 50 nM final concentration using INTERFERin™ (Illkirch, France) as the transfection reagent according to the manufacturer’s instruction. A scrambled siRNA was applied as a negative control. Cells were harvested 72 h later for gene expression analysis by western blot.

### Western blotting

Protein extracts were separated by electrophoresis in a sodium dodecyl sulfate-polyacrylamide gel (Invitrogen, Camarillo, CA, USA) and transferred onto polyvinylidene fluoride membranes (Millipore, Billerica, MA, USA) for immunoblotting. The membranes were hybridized with a primary antibody at 4 °C overnight followed by incubation with a secondary antibody for 1 h at room temperature. β-actin (Santa Cruz, Shanghai, China) was used as the loading control. Signals were visualized using a chemiluminescent detection system (Thermo Scientific, Rockford, IL, USA) and exposure to the film. The antibodies used for western blotting is anti-MTDH (Cell Signaling, Shanghai, China).

### Cell viability, colony formation and apoptosis assays

For clonogenic survival assays, cells plated into petri dishes (60 mm × 15 mm) (2,500 cells per 10-cm plate) and grown for 7–10 days, ﬁxed, and stained by crystal violet. The number of colonies (>50 cells) was counted. For the MTT assay, cells were plated into 96-well plates. Cell growth was monitored 96 h after transfection by the MTT [(4, 5-dimethyl-thiazol-2-yl)-2, 5-diphenyltetrazolium] (Sigma) assay. **Apoptotic and dead cell counts were performed using FITC-labeled annexin V and PI staining (BD Pharmingen, San Diego, CA, USA), followed by flow cytometry (Becton Dickinson, San Jose, CA, USA). Briefly, the cells were gently vortexed and resuspended in binding buffer at a concentration of 3 × 10^6^/ml, and then 100 μl of cell suspension was added to 5 μl of annexin V-FITC and 10 μl of PI and mixed for 15 min in the dark at room temperature. Next, 400 μl of PBS was added to the solution. A FACScan instrument (BD Biosciences) was used to count the cells (1 × 10^3^) at an excitation wavelength of 490 nm. BD FACSDiva 4.0 Software was used for data collection and processing.**

### Reverse transcription and quantitative real-time PCR

Total RNA was extracted from NB tissues and NB cells with Trizol Reagent (Invitrogen, CA, US), according to the manufacturer’s instructions. All RNA extractions were carried out in a sterile laminar flow hood using RNase/DNase-free laboratory ware. The integrity of total RNA was verified by nanodrop (Celbio Nanodrop Spectrophotometer nd-1000). The single-tube TaqMan miRNA assay (Applied Biosystems, CA, US) was used to detect and quantify mature miR-145 and MTDH, using ViiA7 RT reader (Applied Biosystems, CA, US); the protocol was performed for 40 cycles at 95°C for 3 min, 95°C for 15 s, and 60°C for 30 s. miR-145 and MTDH expression was normalized on U6, and then expressed as fold change (2^ΔΔCt^).

### In vivo *orthotopic mouse model*

All mice used in this study were housed and maintained according to guidelines set by the affiliated hospital of Qingdao University on Human Care and Use of Laboratory Animals. The mouse study was approved and supervised by the affiliated hospital of Qingdao University. SH-SY-5Y cells stably infected with either lentivirus containing empty lentiviral vector (SH-SY-5Y -LVEV) or with lentivirus containing a miR-145 overexpressing lentiviral vector (SH-SY-5Y −145 LV) were injected in the back of the 7- to 8-week-old female BALB/c nu/nu mice. Tumor growth was monitored with a caliper 3 times a week and tumor volume was measured by caliper and calculated as length × width × depth × (pi/6). The mice were sacrificed after treatment for 5 weeks, and the neoplasms were fixed in 4% paraformaldehyde. Tissue sections were stained by H&E and IHC.

### Immunohistochemistry

To assess cell proliferation, 5 µm thick formalin-fixed paraffin-embedded sections were stained for Ki-67 (1:400 dilution; Lab Vision, Thermo Fisher Scientific). MTDH and Ki-67 positive cells were counted in three random fields per slide and five slides per group were analyzed at 200x magnification. To assess apoptosis, fresh-frozen sections of tumor tissues were stained by terminal deoxynucleotidyl transferase-mediated deoxyuridine triphosphate nick-end labeling (TUNEL; green; Promega) and counterstained with Hoechst 1:10,000 (10). TUNEL-positive cells were counted in three random fields per slide and five slides per group were analyzed by fluorescence microscopy at 200x magnification.

### Statistical analysis

All Statistical analyses were performed using GraphPad Prism 5.0 by unpaired Student’s t test or one-way analysis of variance followed by Dunnett’s posttest. p < 0.05 was considered statistically significant.

## Results

### MiR-145 expression is significantly associated with event free and overall survival in neuroblastoma

**Figure 1.**
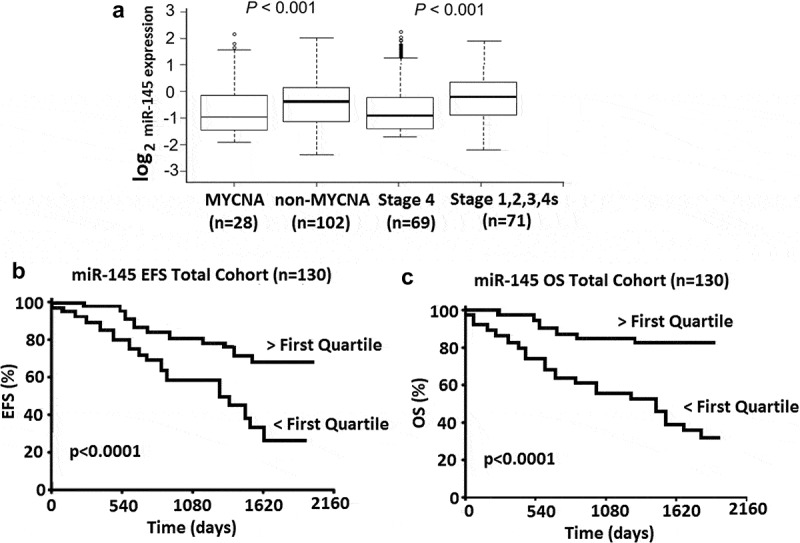
MiR-145 expression is significantly associated with risk and survival in a neuroblastoma cohort (n = 130). (a), Box and whiskers plots represent the expression of miR-145 in a cohort of 130 primary neuroblastoma tumors. Samples are grouped according to known risk factors and expression compared in each of; MYCN amplified versus Non MYCN amplified or Stage 4 versus INSS Stage 1,2,3,4S. Statistical differences in median expression were analyzed using two sided Mann–Whitney U tests. (b), Kaplan-Meier plots for event free survival (EFS) in 130 neuroblastoma patients. (c), Kaplan-Meier plots for overall survival (OS) in 130 neuroblastoma patients. P values were obtained using log-rank test.

Analysis of miR-145 expression levels in 86 primary diagnostic neuroblastoma samples revealed significantly lower expression of miR-145 (based on median expression) in patients with known higher risk prognostic factors including MYCN amplification (MNA) [23] and INSS Stage 4 disease [24] (Figure 1(a)). **The first quartile is referred to as the 25th percentile. More than 25th percentile (> first quartile) of miR-145 expression (high expression)** was significantly associated with both improved event free survival (EFS 5 year 68% vs 26%) (Figure 1(b)) and overall survival (OS 5 year 78% vs 36%) (Figure 1(c)), indicating a potential tumor suppressor role in neuroblastoma.

### *MiR-145 overexpression inhibits cell growth and induced cell apoptosis* in vitro

**Figure 2.**
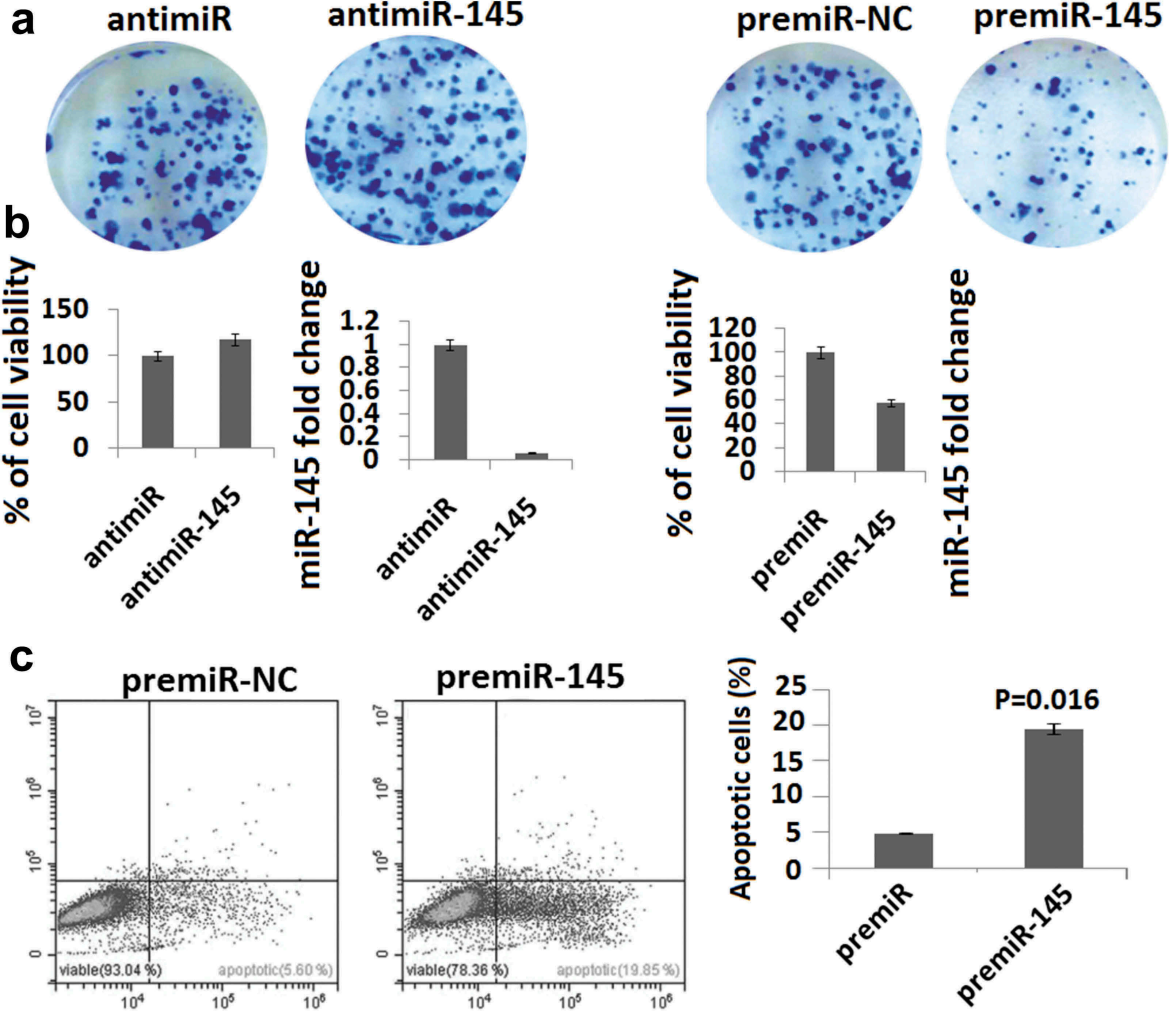
MiR-145 overexpression inhibits cell growth and induces apoptosis. (a), SH-SY-5Y cells were transfected antimiR-145 or premiR-145 or premiR negative control (NC), or LNA scrambled antagomiR (Scr antimiR). Cells were grown for 10 days, ﬁxed, and stained by crystal violet. (b), SH-SY-5Y cells were transfected with antimiR-145 or premiR-145 or premiR negative control (NC) and then plated out in 96-well plates. Cell growth was measured by MTT assay at 72 h. P values were obtained by using a two-sided Student t test. miR-145 levels are shown as measured by qPCR 24 h after transfection of LNA oligonucleotides or premiR negative control (NC). (c), Cell apoptosis was detected by Annexin-V staining and propidium iodide (PI) exclusion using the FITC Annexin-V Apoptosis Detection Kit.

To examine the role of miR-145 on cell growth, we tested its effect on the transformation properties of SH-SY-5Y cell lines. After transfection of antagomiR-145 we observed that SH-SY-5Y cell lines formed larger colonies than controls (Figure 2(a)). However, transfection of miR-145 precursor, but not scrambled oligonucleotide, formed smaller colonies than controls (Figure 2(a)). As shown in Figure 2(b), treatment by antagomiR-145, but not scrambled oligonucleotide, increased cell growth in SH-SY-5Y cell lines by 18%. By contrast, transfection of miR-145 precursor, but not scrambled oligonucleotide, reduced cell growth in SH-SY-5Y cell lines by 40% (Figure 2(b)). Consistent with these results, a significant increase in apoptosis activity was observed in SH-SY-5Y cells following miR-145 transfection, as determined by Annexin V/PI staining and FACs analysis at 72 h following miR-145 transfection (Figure 2(c)).

### MiR-145 inhibits tumorigenesis in vivo

**Figure 3.**
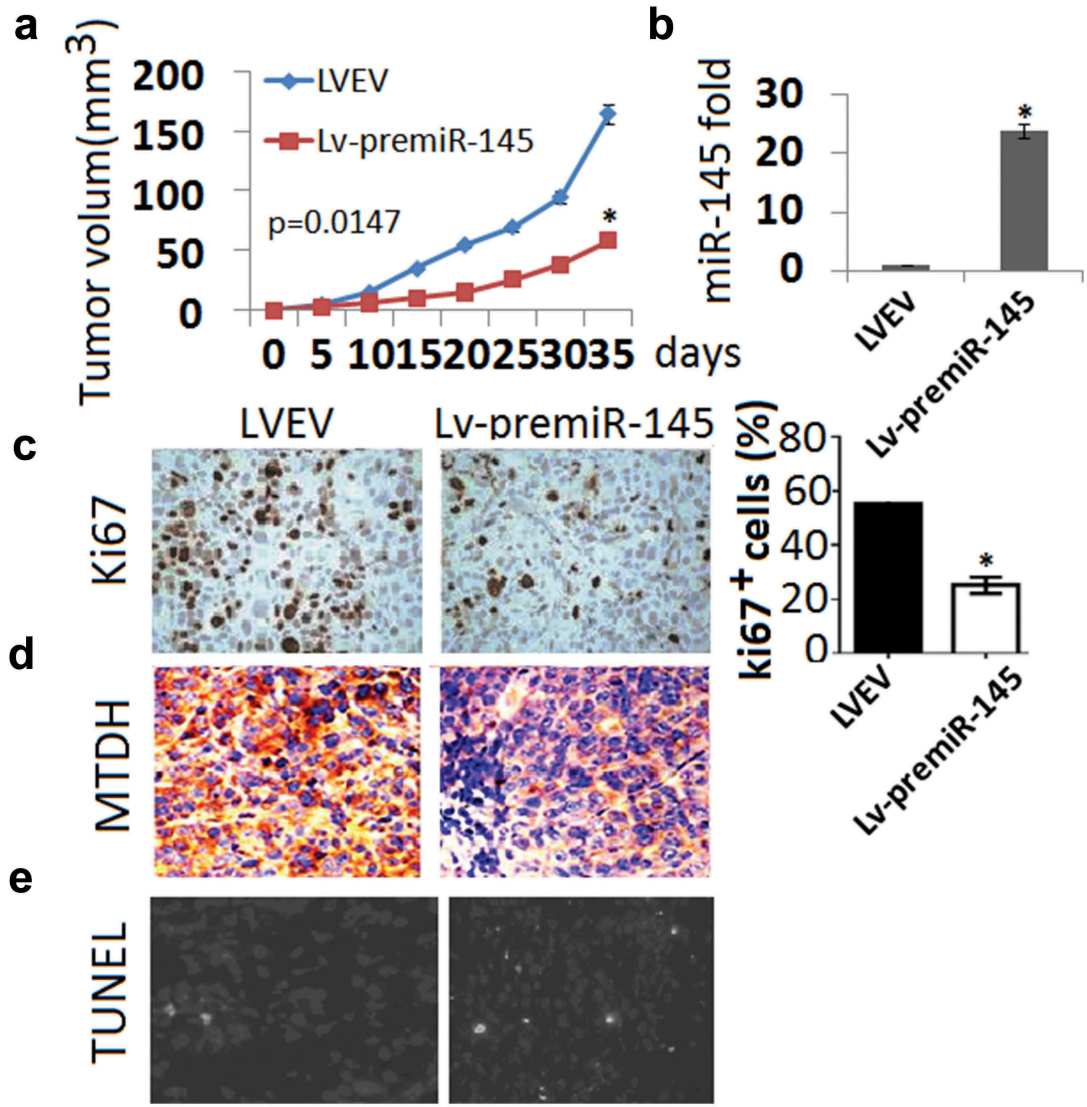
MiR-145 overexpression inhibits tumor growth in xenografted ovarian models. (a), Changes in tumor volume between SH-SY-5Y cells carrying miR-145 group and SH-SY-5Y cells carrying empty vector group. Data are shown as mean tumor volume ± SD. (b), Expression of miR-145 in miR-145 group and empty vector group; (c), IHC staining assay for ki-67 in two groups; (d), IHC staining assay for MTDH in two groups E, TUNEL staining in two groups.

These data prompted us to investigate the role of both miR-145 in tumor development. SH-SY-5Y cells were infected with a retrovirus that overexpresses miR-145 or with an empty vector, and assayed for their ability to form tumors in vivo. All constructs were **subcutaneous** (s.c) injection in a limited number of animals (n = 4). In mice injected with SH-SY-5Y cells carrying miR-145, the average tumor volume was significantly decreased when compared with empty vector. This difference was statistically signiﬁcant (P = 0.0147, Student’s t test; Figure 3(a)). Expression levels of miR-145 constructs were monitored and found to be >20-fold above the endogenous levels in transduced cells (Figure 3(b)). Histopathological analysis demonstrated decreased cell proliferation and MTDH expression and increased cell death, as shown by Ki-67 (Figure 3(c)), MTDH (Figure 3(d)) and TUNEL staining (Figure 3(e)), respectively, in miR-145-overexpressing tumors.

### miR-145 directly suppresses MTDH

**Figure 4.**
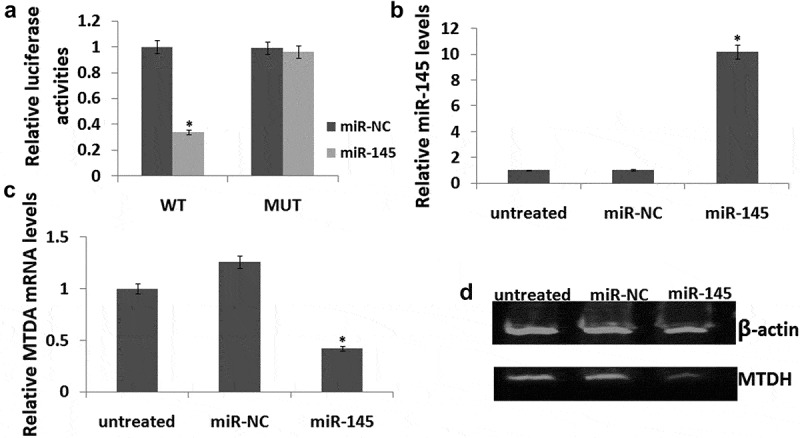
MTDH is the direct target of miR-145. (a), Relative luciferase activity of SH-SY-5Y cells after co-transfection with wild-type (WT) or mutant MTDH 3ʹ UTR reporter genes and miR-145 or miR-NC. The relative luciferase activity was normalized to Renilla activities. (b), Co-transfection with wild-type (WT) or mutant MTDH 3ʹ UTR reporter genes and miR-145 or miR-NC. miR-145 expression were detected by qRT-PCR.(c), MTDH mRNA expression levels was detected by qRT-PCR. (d), The MTDH protein levels were analyzed by Western blotting. The results are presented as mean ± SE (n = 3). *p < 0.05.

To elucidate whether MTDH is directly repressed by miR-145, SH-SY-5Y cells were co-transfected with the MTDH 3ʹ-UTR and miR-145 mimic or miR-NC. Luciferase reporter assay results showed that co-transfection with miR-145 obviously reduced the luciferase activity of the MTDH 3ʹ-UTR wild-type (WT) reporter gene (Figure 4(a)), and increased miR-145 expression (Figure 4(b)). Next, we detected the expression level of MTDH after miR-145 overexpression by qRT-PCR (Figure 4(c)) and western blotting (Figure 4(d)) in SH-SY-5Y cells. We noticed that the MTDH mRNA and protein levels were significantly decreased by miR-145 mimic compared with the miR-NC (Figure 4(c,d)).

### Targeting MTDH inhibited cell proliferation and induced cell apoptosis

**Figure 5.**
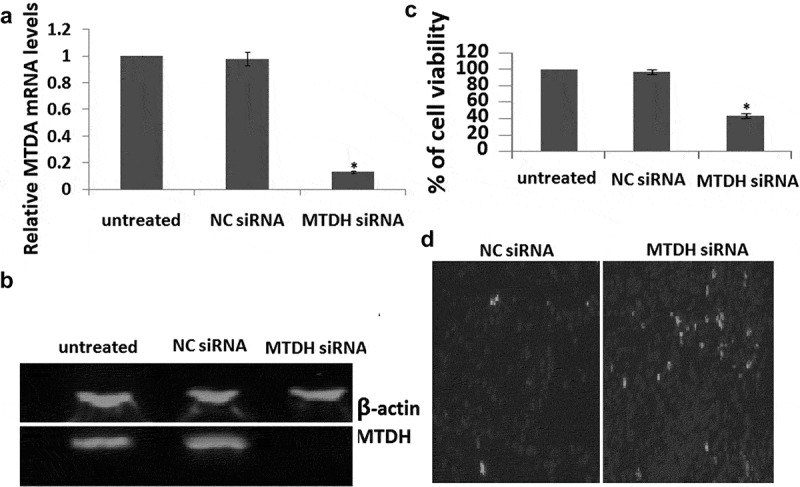
MTDH is involved in SH-SY-5Y cell proliferation (a), MTDH expression levels after siRNA transfection were detected by qRT-PCR. (b), The MTDH protein levels were analyzed by Western blotting in cells expressing siRNA. (c), Cell growth was measured by MTT assay at 72 h transfection. (d), TUNEL staining in two groups. The results are presented as mean ± SE (n = 3). *p < 0.05.

We silenced MTDH to assess whether MTDH downregulation inhibits SH-SY-5Y cell proliferation and induced apoptosis. The results showed that targeting MTDH (Figure 5(a,b)) decreased cell proliferation and induced cell apoptosis which was the **similar to** miR-145 overexpression (Figure 5(c,d)).

## Discussion

The dysregulation of miRNAs is a key mechanism involved in the pathogenesis of neuroblastoma, with several tumor suppressor miRNAs having been identified. Here we determined that expression of miR-145, another potential tumor suppressor in neuroblastoma, was significantly lower in high-risk MYCN amplified tumors and that lower miR-145 expression was associated with worse EFS and OS in our patient cohort. Similarly, miR-145 expression has been associated with improved patient survival in other cancers, including esophageal squamous cell carcinoma and cervical cancer, suggesting a wider tumor suppressor role for miR-145 [25,26]. Ectopic expression of miR-145 decreased breast cancer cell proliferation and increased apoptosis in colorectal cancer cell lines [27,28]. Similarly, we observed a significant increase in apoptosis in our neuroblastoma cell lines following over-expression of miR-145 in vitro.

To date, the characterization of miR-145 function has not been extensive, although several targets have been identified with a key role in cell cycle and survival pathways, including p53, N-RAS and VEGF, and ADAM17 [29–31].

MiR-145 could inhibit cell invasion and migration by targeting TWIST in Colorectal Cancer [32], TGFBR2/Smad3 axis in bladder cancer [33], phospholipase C epsilon 1 in esophageal squamous cell carcinoma [34], ADAM19 in glioblastoma [35], and TGF-β1 in breast cancer [36].

Metadherin (*MTDH*; also called *AEG1, LYRIC*) as a pro-metastasis gene that resides in 8q22, a frequently amplified genomic locus linked to poor relapse-free survival of breast cancer [37]. Notably, overexpression of MTDH is observed in more than 40% of primary tumors and is an independent poor-prognosis factor [38]. Recent studies using cell culture or xenograft models have implicated MTDH in several cancer-related processes, including proliferation, cell death, invasion, and angiogenesis [37], although the underlying mechanistic understanding of MTDH in these processes remains limited to date.

Pan et al. reported that MTDH functions as an oncogene in PCa and the inhibition of MTDH by miR-145-5p or miR-145-3p suppressed the growth and metastasis of PCa cells [39]. Mataki et al. reported that both strands of miR-145, miR-145-5p andmiR-145-3p are functional and play pivotal roles as antitumor miRNAs in lung SCC by targeting MTDH [40]. In the present study, we determined that miR-145 directly targets and inhibits MTDH protein expression in neuroblastoma cell lines, resulting in increased apoptosis and growth inhibition in neuroblastoma cells, consistent with previous findings that miR-145 inhibition of MTDH produced a more significant increase in apoptosis in lung SCC cells [40]. Furthormore, the silencing of MTDH alone also inhibited the cell viability of SH-SY-5Y cells. *In vivo*, miR-145 overexpression inhibits neuroblastoma growth and induced cell apoptosis, followed by downregulation of MTDH. It is suggested that miR-145 overexpression inhibits neuroblastoma growth by direct downregulating of MTDH.

## Conclusion

In conclusion, miR-145 was low expressed in high-risk MYCN amplified (MNA) neuroblastoma, and low miR-145 expression was associated with worse survival in patients with neuroblastoma. Enforced miR-145 inhibited neuroblastoma growth *in vitro* and *in vivo* by targeting MTDH, thus providing a potential target for the development of an microRNA-based approach for neuroblastoma therapy.

## Highlights

1. miR-145 expression was significantly lower in high-risk MYCN amplified (MNA) neuroblastoma
2. Low miR-145 expression was associated with worse EFS and OS in patients with neuroblastoma
3. Enforced miR-145 reduced cell viability and increased apoptosis in neuroblastoma cells in vitro.
4. MTDH is a direct target for miR-145 in neuroblastoma
